# New measurement method for long-term oral complications after harvesting buccal mucosa grafts for urethroplasty

**DOI:** 10.1186/s13005-025-00526-5

**Published:** 2025-07-09

**Authors:** S. Farahzadi, M. Buckova, M. Von Witzleben, T. A. Schröder, G. Lauer, P. Korn

**Affiliations:** 1 Department of Oral and Maxillofacial Surgery, Faculty of Medicine Carl Gustav Carus, University Hospital, Technische Universität Dresden, Dresden, Germany; 2 Centre for Translational Bone, Joint and Soft Tissue Research, University Hospital Carl Gustav Carus, Faculty of Medicine Carl Gustav Caru, Technische Universität Dresden, Dresden, Germany

**Keywords:** Buccal mucosa transplantation, Urethroplasty, Long-term complications, Donor site morbidity

## Abstract

**Background:**

Patients undergoing oral mucosa harvesting for urethroplasty may experience several challenges during recovery like scarring or changes in oral sensitivity, which can lead to long-term discomfort. In this retrospective study long-term donor site complications after harvesting of oral mucosa for urethroplasty were evaluated and a new measurement method for oral volume differences between the non-operated and operated sides was applied.

**Methods:**

Thirty adult male patients who underwent urethroplasty with buccal mucosa grafting were included. At a median of 43 months after surgery, a standardized questionnaire was used, and clinical examinations were conducted to measure the postoperative elasticity of the buccal mucosa. This measurement compared the non-operated side with the operated side. Additionally, we examined descriptive statistics and the influence of smoking status, diabetes mellitus, immunosuppression, alcohol consumption, and graft size.

**Results:**

In total, 36% of the patients reported persistent subjective postoperative impairments, such as tightness in the oral cavity or numbness. In all patients, a difference in buccal volume was observed between the operated side and the non-operated side. This volume difference ranged from 3 to 15 ml (mean 8.10 ml, SD ± 3.4; *p* < 0.001). As the size of the harvested transplant increased, the postoperative buccal volume difference also increased significantly (*p* < 0.001). In one patient, follow-up surgery was required due to the severity of scarring. The presence of diabetes mellitus, immunosuppressive medication, smoking status, and alcohol consumption had no statistically significant effect on postoperative buccal elasticity or mouth opening.

**Conclusions:**

The use of buccal mucosa grafts for urethroplasty is an established procedure in urology, but the oral harvesting procedure can lead to scarring within the buccal area, which is associated with a statistically significant decrease in buccal volume compared with the non-operated site. The volume analysis was performed by applying a new measurement method, which enables, for the first time, the quantification of oral donor site morbidity.

## Introduction

Various soft tissue grafts, like genital skin grafts or extragenital grafts such as oral mucosa, can be used for the treatment of acquired or congenital defects of the male urethra [1]. The application of buccal mucosa grafts has become a common and popular replacement method in urethroplasty since it was described by El-Kasaby et al. [2]. Buccal mucosa is particularly suitable due to its high vascularity, ease of harvesting, and good long-term results [3–5]. There are also alternative oral donor sites, such as the lingual mucosa [6] or labial mucosa [7]. The opening rates of the urethra after using different mucosa grafts for urethroplasty do not differ. However, buccal and lingual mucosa grafts are associated with distinct donor site morbidities [1]. Their use is often favored over other graft types—such as genital or extragenital skin grafts—largely based on accumulated clinical experience. According to the European guidelines for urethroplasty oral mucosa grafts are recommended [1]. Penile skin grafts receive only a weak recommendation and are suggested only when buccal or lingual mucosa grafts are unavailable or declined by the patient [1]. Alternative donor sites, including postauricular skin, abdominal skin or colonic mucosa, should only be considered when the above-mentioned grafts cannot be used, mainly due to the limited surgical experience with these rarely used grafts for urethroplasty [1].

Few studies have focused on oral complications after the harvest of buccal mucosa for urethroplasty. A limitation in mouth opening has been described as a common long-term complication. Dublin et al. reported a narrowing of the oral cavity in nearly one-third of patients after an average of 20.9 months [8]. Another study reported that the incidence of persistent mouth-opening restrictions was 9% [9]. However, one study also described no post-surgery complications with mouth opening or tightness of the oral cavity [10]. Additionally, patients often report on numbness and postoperative pain as frequent complications [5, 11].

The aim of the following study was to evaluate donor site morbidity after harvesting buccal mucosa grafts with respect to postoperative buccal elasticity and mouth opening limitations. Additionally, the patients received a questionnaire evaluating subjective donor site morbidity, and the impact of general disease was analysed.

## Methods

### Patient inclusion and exclusion criteria

This retrospective, monocentric cohort study was approved by the ethics committee of Technische Universität Dresden, Germany (BO-EK-126032024) and the study followed the Declaration of Helsinki- ethical principles for medical research involving human participants. Adult, male patients who underwent urethroplasty with application of one buccal mucosa graft at the University Hospital Carl Gustav Carus Dresden, Germany, between 2016 and 2023 were included in the study. Thirty of 77 patients agreed to participate in the study, which included a questionnaire (Table 1) and a clinical examination. Exclusion criteria included age under 18 years, female sex, bilateral oral mucosa harvesting, and inability to provide informed consent. Patients with contraindications to oral mucosa harvesting identified preoperatively were excluded for surgical reasons prior to the retrospective analysis. These contraindications included, for example, oral mucosal pathologies such as lichen planus or a history of radiotherapy to the head and neck region. Edentulism was not defined as an explicit exclusion criterion. However, all patients enrolled in the study had either full natural dentition or prosthetic restorations extending from the second molar to the second molar in both the maxilla and mandible.

**Table 1:** Questionnaire

**Last Name and First Name**:			
**Date of birth**:			
**Patient ID**:			
**Control date**:			
**Operation date**:			
**Clinical photo**:			
**Donor site**: **Unilateral/ Bilateral**:	○ Left ○ Right		
**Size of the mucosa graft**:			
**Smoking Status**:	○ Yes ○ No If yes, how many cigarettes on average per day:		
**Alcohol consumption**:	○ occasional ○ regular If yes, how much:		
**Comorbidities**:			
**Subjective impairment**:	○ Yes ○ No If yes, which:		
**Mouth opening (distance between incisal edges)**:			
**Mouth Closure (e.g.**,** whistling)**:			
**Tightness in the oral cavity (buccogingival spaces)/ subjective**:	○ Yes ○ No		
**Tightness in the oral cavity (buccogingival spaces)**: how many ml:			
**Numbness**:	○ Yes ○ No If yes: Left/ Right Previous numbness:		
**Dry mouth**:	○ Yes ○ No		
**Dental prothesis**:	○ Yes ○ No If yes, have you had problems with the dental prosthetics since the operation:		
**Cancer after the operation at the donor site**:	○ Yes ○ No If yes, when:		
**Oral mucosa assessment (e.g.**,** scar)**:			
**Second operation for improvement of complications (e.g.**,** scar correction)**:	○ Yes ○ No If yes, when and where:		
**Would you be willing to undergo another cheek mucosa harvest procedure?**	○ Yes ○ No		
**Success of the Urethroplasty**:	○ Yes ○ No If no, have you been operated on again:		

### Buccal mucosa grafting technique

**Fig. 1:**
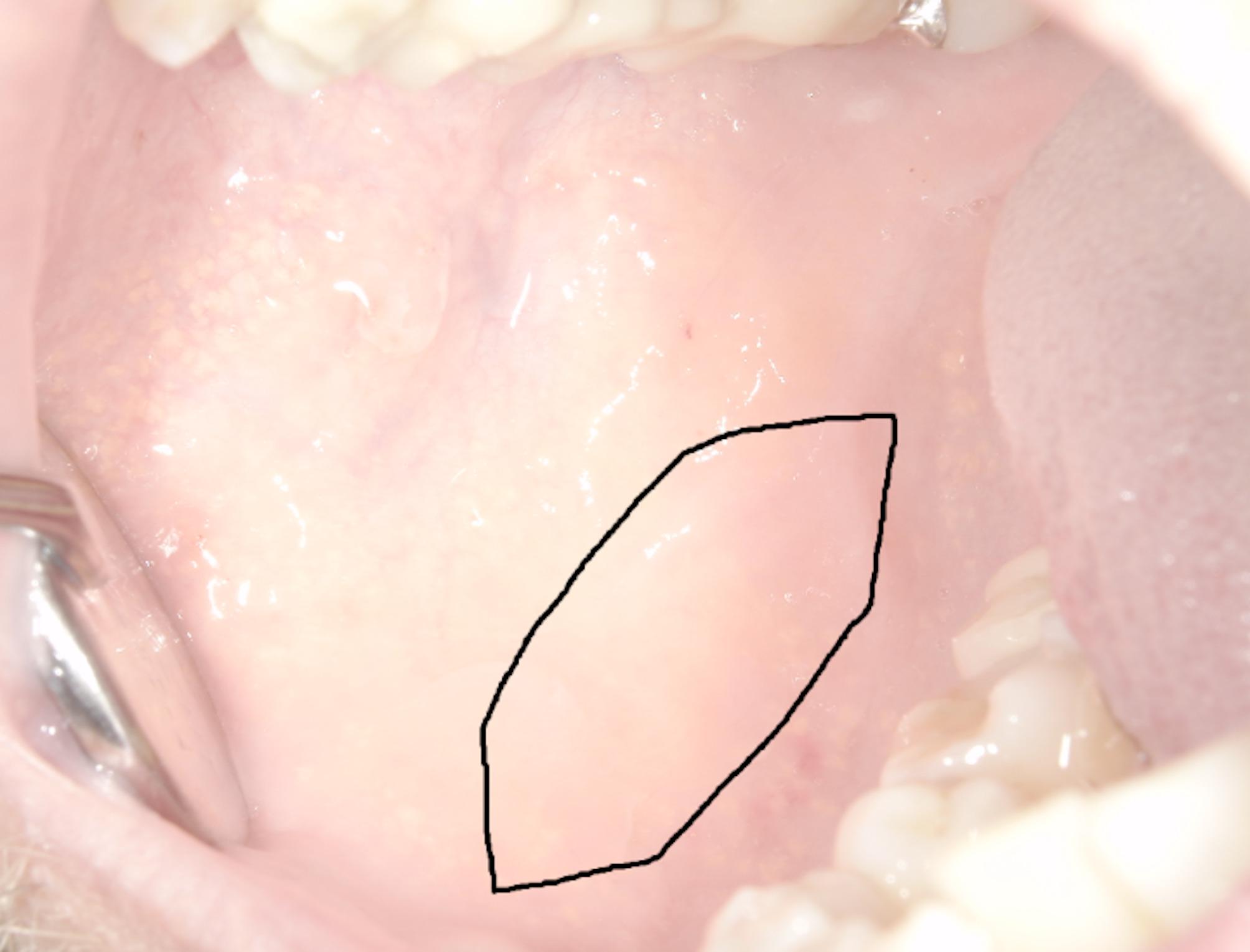
Example of a harvesting site for buccal mucosa grafts within the area of non-keratinized mucosa

Urethroplasty is performed under general anesthesia. First the urethra is prepared by the urologists. The urethral defect is determined, and the required size of the mucosal graft must be specified. Selection of the harvesting site is primarily based on clinical judgment by the maxillofacial surgeon and the required graft size. Subsequently the non-keratinized mucosa of the inner cheek is marked to the appropriate size and shape (Fig. 1). The exact localization of the harvesting site within the planum buccale is determined by both the anatomical characteristics of the individual and the requirements of the recipient site. Nonetheless, graft harvesting is consistently performed outside the zone of attached mucosa and with careful avoidance of critical structures, including the mental foramen and the parotid duct. The thickness of the harvested graft varies according to the patient’s tissue characteristics and the specific demands of the planned surgical procedure. In urethroplasty, for instance, thin grafts comprising only the mucosal layer are preferred.

While the patient is under general anesthesia for the urological procedure, the maxillofacial surgeon concurrently harvests the mucosal graft from the oral cavity. Prior to graft harvesting, a local anesthetic is administered to the designated intraoral site to ensure adequate regional analgesia and hemostasis. The mucosa is then carefully dissected along the cheek via scalpel and gently lifted, ensuring that the underlying muscle and connective tissue are not damaged. The graft is held only at one corner using tweezers, and the rest of the graft is untouched to avoid damage. To prevent significant bleeding, electrocoagulation is used if necessary. After successful harvesting, the mucosal edges are adapted to the underlying tissue, to prevent bleeding. These edges are not adapted to each other, as this would significantly reduce the surface area resulting in more intense scarring. Urethroplasty is then performed using the harvested buccal mucosa graft. No covering of the donor site was performed due to the standardized surgical procedure. The European guideline leaves this decision to the treating surgeon [1]. Postoperative care consisted of a soft diet for 14 days, with recommendations for smoking and alcohol cessation. Patients with dentures were permitted to wear them provided they did not interfere with the surgical site and could be safely inserted and removed for hygiene purposes. Oral hygiene practices, including twice-daily tooth brushing, were maintained by the patients independently, as per their preoperative routine. All patients received intravenous antibiotics prescribed by the urologist during hospitalization, typically for five to seven days. Analgesic medication was administered according to individual needs, following the WHO pain management guidelines. Prior to discharge, intraoral wound healing was assessed by oral and maxillofacial surgeons. While the postoperative regimen was generally consistent across patients, individual factors—such as denture use and adherence to medical recommendations—may have influenced outcomes.

### Questionnaire and analysed medical history

The questionnaire (see supplement) used was completed during follow-up and included variables such as patients age, date of operation, date of follow-up, size of the buccal mucosa graft and success of urethroplasty. Information regarding the date of surgery and graft size was obtained from the hospital’s internal documentation program (Orbis, DH Healthcare GmbH, Bonn, Germany). Furthermore, subjective impairments, such as numbness, dry mouth, or tightness of the oral cavity, were evaluated. Finally, patient-reported information regarding smoking status, alcohol consumption, and relevant comorbidities was documented. This included the presence of diabetes mellitus, use of immunosuppressive medications, anticoagulant or antiplatelet therapy, and a history of malignancy, with or without radiotherapy to the graft harvesting site.

#### Clinical examination

**Fig. 2:**
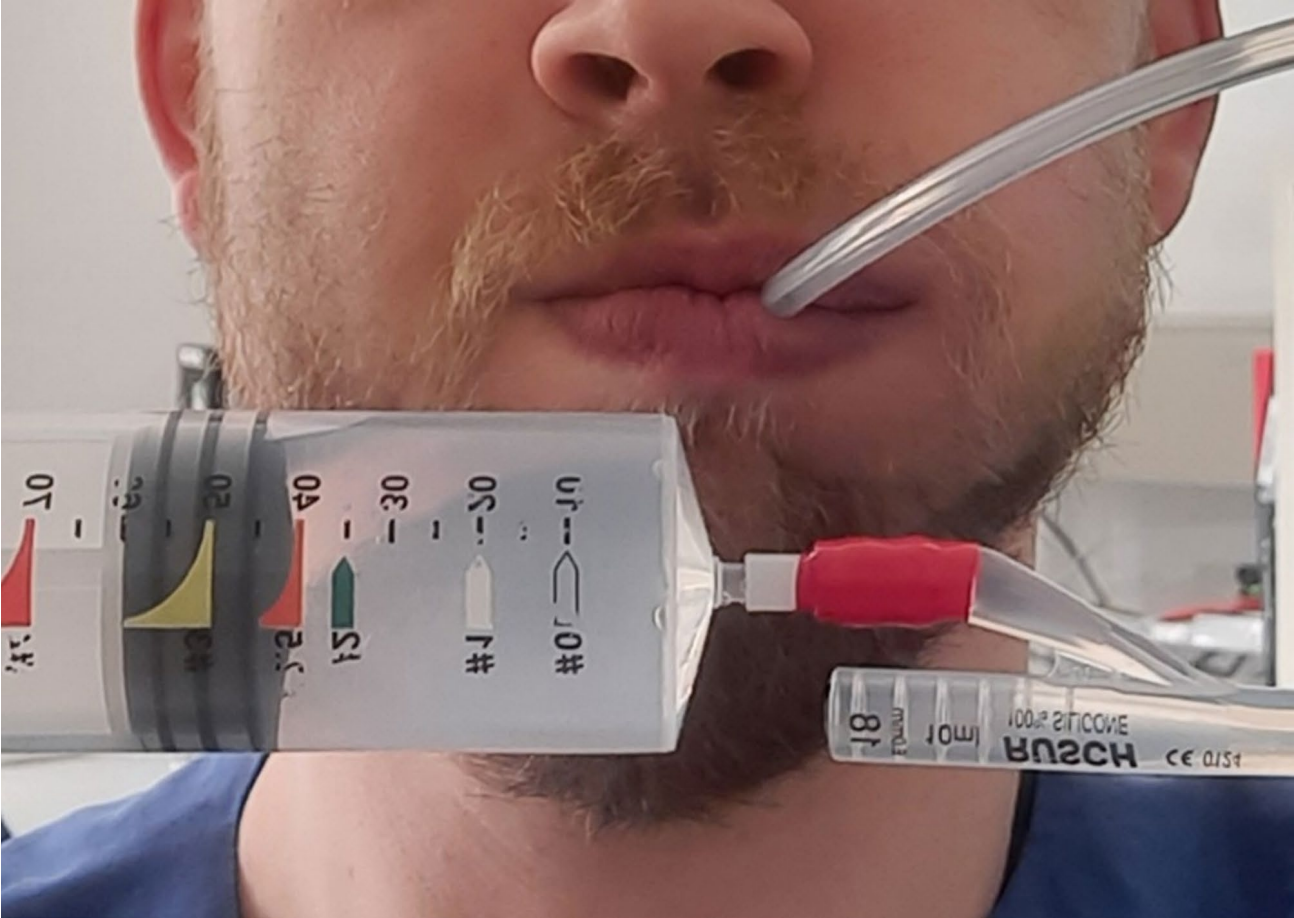
Measurement of the extension of the cheek with a Foley catheter and sterile water

During the clinical examination, the incisal edge distance was measured to assess mouth opening, and the stretchability of the buccal mucosa was examined with a side-by-side comparison. For this purpose, a Foley catheter of size 18 Charrière was inserted between the cheek and the dental arch with the teeth closed and filled with sterile water to measure the expansion of the cheek (Fig. 2). A Foley catheter was inserted into the oral vestibule with the tip advanced as far posteriorly as possible, reaching the region of the posterior molars. Due to interindividual anatomical variability, recording the exact insertion depth was deemed non-informative, as the measurements would not be reliably comparable across patients. The measurement was terminated upon the sensation of tension in the cheek or when the corner of the mouth passively opened due to the catheter’s filling. The same measurement conditions were maintained for the contralateral side. The measurements were repeated three times, and the mean value was subsequently calculated. All patients either had a full dentition or a complete dental arch provided by prosthesis. Patients were instructed to keep their teeth closed until measurements were completed.

Before intraoral application, the maximum load capacity of a Foley catheter of size 18 Charrière was tested, and an extraoral pilot measurement was conducted. The maximum filling volume was measured at 75 ml. In a healthy volunteer with a height of 183 cm and a weight of 98 kg, we measured the maximum expansion of the cheek with 40 ml of sterile water in the Foley catheter intraorally. Due to the stiff material properties of the Foley catheter, displacement of the balloon into the posterior oral cavity is unlikely and was not observed during the measurements. The risk of the Foley catheter bursting during intraoral measurements was not present because of the significantly smaller filling volumes.

Additionally, the incisal edge distance was examined to assess mouth opening, lip closure with lip sealing and the tip of the lips. Further analyses included the volume of the operated and non-operated sides, numbness, dry mouth, dentures, malignancies at the donor site, and follow-up surgeries due to possible local complications after mucosal graft harvesting.

### Statistics

SPPS^®^ (IBM SPSS Statistics for Windows, Version 30.0. IBM Corp, Armonk, NY, USA) was used for statistical analysis.

A Wilcoxon signed-rank test was conducted to compare the volume differences between the operated and non-operated sides, and the effect size (r) was determined based on the significance (*p* < 0.05). Kendall’s tau correlation coefficient was then used to examine whether the size of the buccal mucosa graft influenced scarring or the volume difference between the operated and non-operated sides. Additionally, the effect of diabetes mellitus, immunosuppression, and smoking on scarring and volume differences between the operated and non-operated sides were determined using the Mann-Whitney U test, whereas the effect of alcohol consumption was assessed using the Kruskal-Wallis test. For diabetes mellitus, immunosuppression, and smoking status, a distinction was made between the presence or absence of the condition, whereas for alcohol consumption, the distinction was made between no consumption, occasional consumption, and regular consumption.

## Results

### Descriptive analysis: patients

The patient cohort was between 23 and 80 years old, with a mean age of 62.9 years (SD ± 13.3) at the time of surgery. The mean follow-up period between surgery and the examination was 43.6 months (SD ± 26.1) (Table 2). All patients underwent primary harvesting of oral mucosa for urethroplasty.

Seven patients (23.3%) reported regular alcohol consumption, 16 patients (53.3%) reported occasional alcohol consumption, and 7 patients (23.3%) did not consume alcohol at all. All patients were asked about their smoking behaviours, and 2 of 30 patients (6.7%) were smokers. Regarding their general medical history, 4 patients (13.3%) reported type 2 diabetes mellitus, and 2 patients (6.7%) received immunosuppressive therapy (Table 3). Nine patients (30%) were on single antiplatelet therapy, and one patient (3.3%) was receiving oral anticoagulation, which was temporarily discontinued for up to two weeks postoperatively. During hospitalization, all patients received thrombosis prophylaxis in accordance with our institutional protocols.

**Table 2:** Patient characteristics 1 (SD: standard deviation)

	Min	Max	Mean
Age (years)	29	80	62.9 (SD ± 13.3)
Follow-up time (month)	11	95	43.6 (SD ± 26.1)
Graft size (cm^2^)	3.8	12	6.5 (SD ± 1.8)
Buccal volume operated side (ml)	12	30	20 (SD ± 4.5)
Buccal volume non-operated side (ml)	20	45	28 (SD ± 5.3)
Incisal edge distance (cm)	3.5	6	4.7 (SD ± 0.6)

**Table 3:** Patient characteristics 2

Objective items:	Yes: *n* (%)	No: *n* (%)
Smoking status	2 (6.7%)	28 (93.4%)
Alcohol	sometimes: 16 (53.3%) regularly: 7 (23.3%)	7 (23.3%)
Diabetes mellitus	4 (13.3%)	26 (86.7%)
Immunosuppressive medication	2 (6.7%)	28 (93.4%)
Single antiplatelet therapy	9 (30%)	21 (70%)
Oral anticoagulation	1 (3.3%)	29 (96.7%)
Prothesis	12 (40%)	18 (60%)
Mouth closure	29 (96.7%)	1 (3.3%)
Malignancy of harvesting side	0 (0%)	30 (100%)
Second operation of harvesting side	1 (3,3%)	29 (96.7%)
Success of urethoplasty	17 (56.7%)	13 (43.3%)
**Subjective items**:		
Subjective impairment	8 (26.7%)	22 (73.3%)
Tightness of oral cavity	8 (26.7%)	22 (73.3%)
Numbness	4 (13.3%)	26 (86.7%)
Dry mouth	3 (10%)	27 (90%)

### Descriptive analysis: Buccal mucosa graft and clinical outcome postoperatively

A left-sided buccal mucosa graft was performed in 20 patients (67%), whereas the other 10 grafts were harvested from the right planum buccale. The size of the graft was documented in the operation protocols in centimeters by centimeters. The calculated graft sizes ranged between 3.8 and 12.0 cm², with a mean of 6.5 cm². The mean incisal edge distance was 4.7 cm (SD ± 0.6). In 29 patients (96.7%), complete mouth closure or lip tip contact was possible.

There was no significant association between patient-reported tightness in the oral cavity and the size of the graft according to the Mann-Whitney U test (*p* = 0,52).

One patient underwent a second surgery for scar correction, and three additional patients chose to undergo scar revision after receiving detailed counselling during the follow-up examination.

40% of our patients wore a removable prosthesis, but no impairments such as injuries caused by the clasps or injuries due to insertion and removal of the prothesis occurred postoperatively. No patient reported on problems wearing a prothesis due to narrowing of the oral cavity. No patient developed malignancies at the donor site.

### Quantification of postoperative donor-site morbidity

**Fig. 3:**
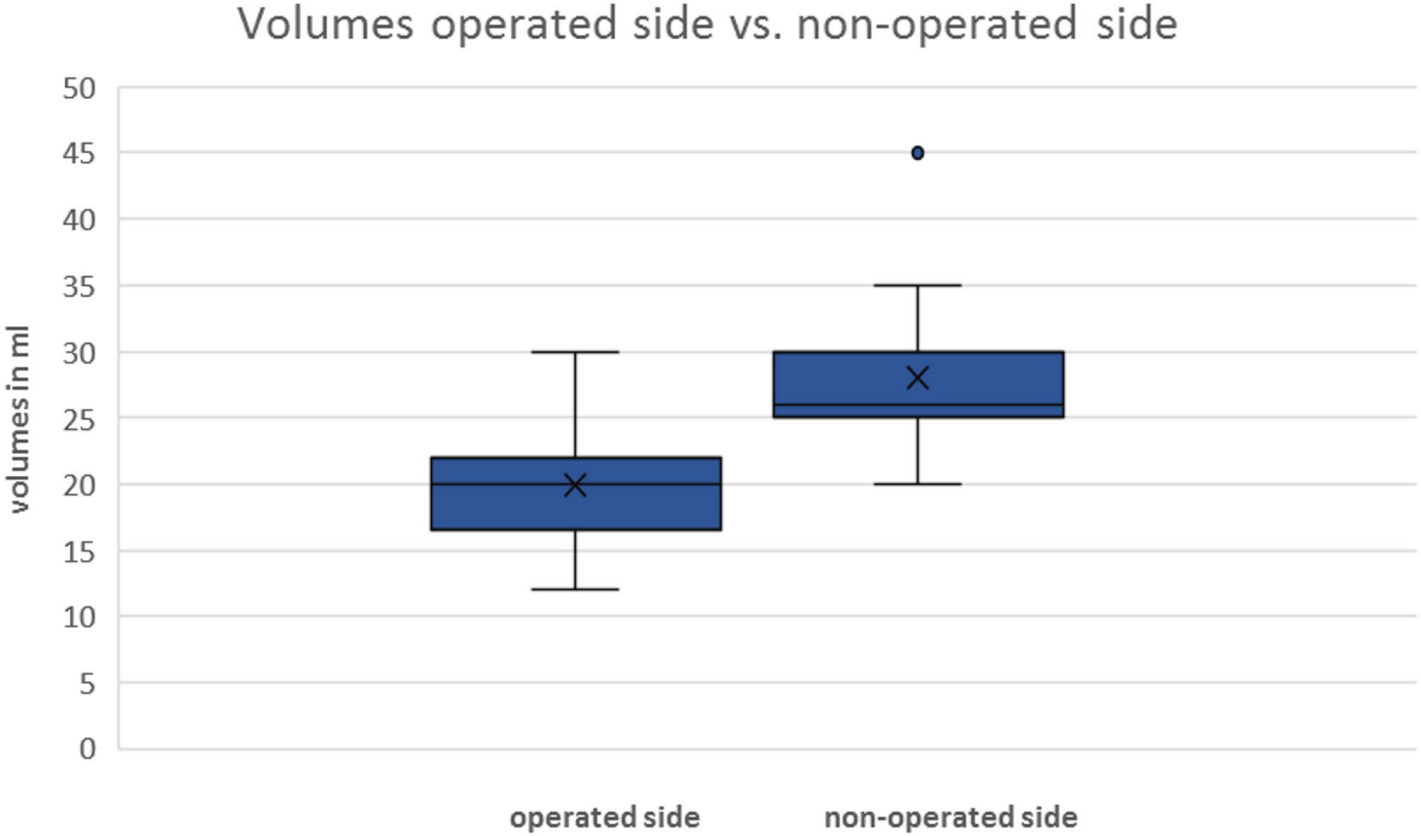
Measured volumes of the operated side (mean: 20 ml; SD ± 4.5 ml) in comparison to those of the non-operated side (mean 28 ml; SD ± 5.3 ml; one outlier: 45 ml). The volume difference was statistically significant

The intraoral volume measured with a Foley catheter on the operated side ranged from 12 to 30 ml, with a mean of 20 ml (SD ± 4.5). The volume of the non-operated side ranged from 20 to 45 ml, with a mean of 28 ml (SD ± 5.3, Fig. 3). The volume differences between the operated and non-operated sides ranged from 3 to 15 ml, with a mean of 8.10 ml (SD ± 3.4). With a p-value of < 0.001 in the Wilcoxon signed-rank test, a statistically significant difference was confirmed. Z was − 4.803. The effect size r =|Z/ √n| was measured, and an r- value of 0.877 was calculated. Using the Kendall-Tau correlation coefficient, the correlation between the size of the transplant and the volume differences between the operated and non-operated sides was confirmed to be significant at a p-value of < 0.001. This suggests that as the size of the transplant increases, there is also a corresponding decrease in measurable volume. The Mann-Whitney U test revealed no significant correlations between the presence of diabetes mellitus (*p* = 0.69), immunosuppressive therapy (*p* = 0.84), or smoking status and the differences in volume between the operated and non-operated sides (*p* = 0.55). The influence of alcohol consumption was analysed via the Kruskal-Wallis test, which also revealed no significant difference (*p* = 0.44). In none of the patients were wound infection or wound dehiscence observed. One patient experienced a local postoperative bleeding at the donor site, which was controlled using local diathermy.

### Subjective results/ questionnaire

Eight patients (26.7%) reported experiencing subjective impairment after surgery. Specifically, they reported sensations of a scar, accidental biting of the donor site, or the accumulation of food remnants in the “pocket” area at the donor site. Eight patients (26.6%) complained of tightness in the oral cavity, which they attributed to a scar. Four patients (13.4%) complained of numbness in the cheek after surgery. Three patients reported dry mouth after surgery. Twenty-seven patients (90%) were willing to undergo surgery again if necessary. Seventeen patients (56.7%) reported that the urethroplasty was successful, and no further surgeries were needed. The remaining patients either needed follow-up procedures, such as urethrotomy, or experienced lasting impairments in urination and were thus followed up as outpatients by an urologist.

## Discussion

The buccal mucosa is currently the most commonly used graft in urethroplasty [1]. Since the technique was first described more than 30 years ago [2], extensive experience has been gained with it. This has resulted not only in good long-term outcomes from a urological perspective [12] but also in low complication rates at the donor site [6]. In contrast to studies that report no complications at the donor site [2, 4, 10], long-term complications are also possible, and patients must be informed about these risks prior to surgery [1]. In the current cohort study, persistent donor site complications were detected. Four patients (13%) reported postoperative numbness at the donor site, 3 patients (10%) reported dry mouth, and another 3 reported the accumulation of food remnants at the site of harvesting. These accumulations are due to pocket formation caused by strong traction forces from the scar tissue. In other studies, the proportion of patients with persistent numbness ranged from 3.7 to 26% [8–11]. With respect to dry mouth, comparable incidences are reported in the literature [9], although in some cases, these incidences are reported to be lower [5, 11]. One of our patients underwent surgery due to persistent and severe tightness in the oral cavity. In the study by Wood et al., one of 49 patients underwent a second operation due to a retention cyst [9]. None of the studies reported patients with repeated accumulation of food remnants at the site of extraction.

In all the patients examined, a difference in the elasticity of the buccal mucosa between the operated and non-operated sides was observed. The larger the harvested mucosal graft was, the greater was the volume difference on the opposite side. With the exception of one patient each, mouth opening and closure were not affected. In the present study, smoking, alcohol consumption or diabetes mellitus had no significant effect on the measured volume differences. We assumed that these factors influence the process of wound healing and scarring, but the number of patients was likely too small to measure the effects of those factors.

To the best of our knowledge, this is the first study applying an indirect measurement method to quantify the elasticity of the buccal mucosa. The observed volume differences at the operated site compared with the non-operated side are attributed to a reduced elasticity of the buccal mucosa after harvesting the mucosa graft. Thus, volume measurement seems to provide an indirect quantification of the extent of scarring. These findings contrast with the approximately 26% of patients who reported a sensation of tightness in the oral cavity. In other studies, the incidence of such a sensation was sometimes lower [9, 11] or even greater [8]. We could not find an association between the size of the harvested graft and patient-reported tightness in the oral cavity. Similarly, we did not find any correlation with the measured volume difference. According to Markiewicz et al., mucosal harvesting from the buccal mucosa is more frequently associated with wound contraction and scarring because of its anatomical relationship to the buccinator muscle [13]. Some prospective studies have investigated whether closure or non-closure of the donor site affects the occurrence of oral complications [1, 14, 15, 16]. Unfortunately, the results are not conclusive, and the European guideline leaves the decision to the treating physician [1]. In all our patients, the operated site was not completely closed; instead, the mucosal edges were sutured to the underlying submucosa.

When reviewing the studies, it becomes apparent that a precise differentiation between a sensation of tightness in the oral cavity and a restriction in mouth opening is not always provided. Some studies have reported only on one of these points [5, 8–11]. To objectively assess the sensation of tightness in the oral cavity, we measured the elasticity of the buccal mucosa using the volume measurement described above. Mouth opening, on the other hand, was evaluated by measuring the distance between the incisal edges. The volume measurement offers the advantages of low cost, ease of implementation and good availability. It allows the quantification of subjectively reported tightness in the oral cavity and differentiation from mouth opening restrictions. In the future, this method could improve the comparability of surgical methods and their effects on the elasticity of the buccal mucosa or the formation of scar tissue.

The retrospective design and the relatively small sample size represent inherent limitations of the present study. However, the study was intentionally designed to address region-specific clinical needs under routine care conditions and without external funding, thereby reflecting real-world clinical practice. Postoperative volume assessment was performed in a standardized and clinically appropriate manner by comparing the operated to the contralateral, non-operated side. Owing to the retrospective nature of the study, preoperative baseline measurements were not available, precluding direct intraindividual comparison. Furthermore, as both patients and the examiner were aware of the intervention site, the potential for observer and response bias cannot be excluded. Balloon catheter inflation was performed based on the patient’s subjective perception of intraoral pressure, permitting individualized adjustment and optimizing patient comfort during the procedure. Notably, the volume of water used for inflation was not disclosed to the patients, thereby minimizing potential bias and reducing the likelihood that subjective responses were influenced by preconceived expectations or reference values. In the present investigation, measurements were primarily based on patient-reported perception and visual assessment, contributing to the simplicity, feasibility, and broad clinical applicability of the method. The use of a single examiner ensured consistency across all evaluations; while this introduces the possibility of systematic measurement bias, it eliminates inter-observer variability.

As a consequence of the study design, the present volumetric assessments relied primarily on subjective patient feedback in combination with clinical inspection. The integration of adjunctive imaging techniques such as three dimensional facial scanning in future research may enhance both objectivity and reproducibility of volumetric assessments. Although the absence of adjunctive imaging techniques may raise concerns regarding measurement precision, it also contributes to the simplicity, cost-efficiency, and broad applicability of the method in routine clinical practice. The primary aim of this study—to evaluate the feasibility of a novel postoperative assessment method—was successfully achieved, with statistically significant outcomes supporting its clinical utility. All patients in the cohort had complete dental arches, as any missing teeth were prosthetically restored. However, the influence of molar absence on the validity and reliability of the measurement approach remains unclear and warrants further investigation. Additionally, operative records did not contain standardized documentation regarding the use of diathermy during buccal mucosa harvesting. Although routinely employed for hemostasis, the extent of diathermy application was not quantified. Given the established influence of intraoperative bleeding and thermal tissue effects on wound healing, the lack of detailed surgical documentation represents a methodological limitation. Nevertheless, as all assessments were performed after clinically complete wound healing, these factors are unlikely to have substantially affected the volumetric outcomes.

Notably, the volumetric asymmetries identified in all patients did not consistently correlate with self-reported symptoms. This discrepancy may be attributed to a postoperative adaptation process, whereby patients gradually become accustomed to changes in oral tissue dynamics, diminishing their perception of tightness or discomfort over time. The limited sample size likely restricted the statistical power to detect associations between objective volume measurements and subjective reports. Furthermore, the timing of postoperative evaluation varied among patients, although all assessments—except one—were conducted beyond the one-year mark, with the remaining case evaluated only two days prior. At this stage, wound healing is considered largely complete, and the variability in follow-up timing is unlikely to have significantly affected the outcomes [17]. The present pilot study successfully demonstrated the feasibility and clinical applicability of a novel method for postoperative volumetric assessment, yielding statistically significant results and providing a robust foundation for future longitudinal research.

To enhance methodological rigor in future investigations, standardized pre- and postoperative assessments by multiple independent examiners are recommended. The integration of advanced imaging modalities, such as three-dimensional facial scanning, may further improve measurement accuracy and reproducibility. A defined postoperative recall protocol with consistent intervals would reduce variability in follow-up timing and allow for more precise analysis of changes in buccal elasticity and mouth opening over time. Given the observed dissociation between objective volumetric findings and subjective symptom reports, future studies should also consider the role of neurosensory adaptation and patient-reported outcome measures. While all patients in the present cohort had complete dental arches through prosthetic restoration, the potential influence of dental status on soft tissue dynamics warrants further examination.

## Conclusions

The newly established intraoral volume measurement method presented here is cost-effective, easy to perform, and highly reproducible. It allows the assessment of the elasticity of the buccal mucosa before and after mucosal graft harvesting, as well as in a side-by-side comparison. The purpose of these measurements is to objectify reported feelings of tightness in the oral cavity and to compare existing surgical methods of mucosal harvesting in terms of the occurrence of this side effect. This provides an opportunity to identify the most suitable method for buccal mucosa graft harvesting and to reduce donor site morbidity for patients who must undergo urethroplasty with the application of a mucosa graft.

## Data Availability

The data that support the findings of this study are not openly available for reasons of sensitivity and are available from the corresponding author upon reasonable request. The data are stored via controlled access data at the Department of Oral and Maxillofacial Surgery, University Hospital and Faculty of Medicine Carl Gustav Carus, Technische Universität Dresden, Dresden, Germany.
